# Seven lessons for interdisciplinary research on interactive digital health interventions

**DOI:** 10.1177/2055207618770325

**Published:** 2018-05-03

**Authors:** Ann Blandford, Jo Gibbs, Nikki Newhouse, Olga Perski, Aneesha Singh, Elizabeth Murray

**Affiliations:** 1 UCL Institute of Digital Health, 4919 University College London, UK; 2 UCL Interaction Centre, 4919 University College London, UK; 3 UCL Centre for Population Research in Sexual Health & HIV, Institute of Global Health, UK; 4 eHealth Unit, Research Department of Primary Care and Population Health, 4919 University College London, Royal Free Hospital, UK; 5 Department of Clinical, Educational and Health Psychology, 4919 University College London, UK

**Keywords:** Human–Computer Interaction; e-health; digital health interventions; interdisciplinarity; multidisciplinary teams; development lifecycles

## Abstract

Research and development for interactive digital health interventions requires multi-disciplinary expertise in identifying user needs, and developing and evaluating each intervention. Two of the central areas of expertise required are Health (broadly defined) and Human–Computer Interaction. Although these share some research methods and values, they traditionally have deep differences that can catch people unawares, and make interdisciplinary collaborations challenging, resulting in sub-optimal project outcomes. The most widely discussed is the contrast between formative evaluation (emphasised in Human–Computer Interaction) and summative evaluation (emphasised in Health research). However, the differences extend well beyond this, from the nature of accepted evidence to the culture of reporting. In this paper, we present and discuss seven lessons that we have learned about the contrasting cultures, values, assumptions and practices of Health and Human–Computer Interaction. The lessons are structured according to a research lifecycle, from establishing the state of the art for a given digital intervention, moving through the various (iterative) stages of development, evaluation and deployment, through to reporting research results. Although our focus is on enabling people from different disciplinary backgrounds to work together with better mutual understanding, we also highlight ways in which future research in this interdisciplinary space could be better supported.

## Introduction

Interdisciplinary research almost invariably poses challenges: of values, assumptions, terminology, methodology and culture. In this paper, we focus specifically on the challenges faced in developing and deploying effective interactive digital health interventions (DHIs), including different views on what is meant by ‘effective’ and ‘implementing.’ Interactive DHIs are interventions designed to improve health that are delivered on a digital platform^1^. Research in interactive DHIs involves two central domains of study: Human–Computer Interaction and software engineering (which we refer to collectively as ‘HCI’) and those originating in biomedical sciences and psychology (which we refer to collectively as ‘Health’). Each of these areas of expertise draws, in turn, on multiple disciplines. The lessons we highlight in this paper are based on years of experience, including frustrations, blockages, incomprehension and (most importantly) discovery, insight and delight. Our intended audience is researchers from the parent subject areas, maybe working together for the first time, and particularly the early career researchers who find themselves steering a course between areas of expertise with different origins, values and established practices. Our aims are to prepare researchers for the challenges and share lessons that we have learned. For simplicity, we present the two cultures as poles while recognising that actual practices are situated between these extremes, and that recent initiatives are developing ‘middle ways’ between these poles. The very point about interactive DHIs is that they draw on multiple disciplines; our focus here is on how to make disciplinary integration effective and to take the best from complementary cultures.

## Background

There is an extensive background literature on developing and evaluating interactive DHIs. In this paper, we restrict ourselves to a targeted review of literature addressing methodological approaches for research in interactive DHIs. We structure this in terms of development lifecycles, evaluation and implementation.

### Development lifecycles

In a review of self-care technologies from an HCI perspective, Nunes et al.^2^ observe that many studies have ‘largely privileged a medical perspective’ (p.33:1) and that many of the papers on methods for designing and evaluating interactive DHIs also have their roots in biomedical sciences. Historically, many interactive DHIs developed within Health research base their approach on the early Medical Research Council (MRC) guidance on developing and evaluating complex interventions^3^. This approach was originally based on the models of developing and evaluating new pharmacological agents for use in health care, and argued that a similarly structured approach is required for complex (non-pharmacological) interventions. In common with drug development, this focused on a sequential model of the development of complex interventions culminating in a randomised controlled trial (RCT) to determine effectiveness. Although a subsequent version of this framework emphasised the need for an iterative approach^4^, there is still a belief among many Health researchers and policy makers that intervention development is a ‘one-off’ event, which should lead to a demonstration of effectiveness through an RCT.

Other researchers have addressed the challenge of developing interactive DHIs by presenting approaches tailored to the needs of Health researchers. For example, Elwyn et al.^5^ present a process map for development of decision support DHIs that involves iterative design and testing ‘until the intervention is deemed accessible and useful’. Focusing on interactive DHIs for behaviour change, Yardley et al.^6^ present the ‘person-based’ approach to intervention development, ‘developed to focus on understanding and accommodating the perspectives of the people who will use the intervention’. They describe this as complementing established theory-based and evidence-based approaches, focusing on qualitative methods to identify psychosocial factors that will enable developers to select design features that are likely to be salient, attractive, and persuasive for the intended users. They also suggest that the person-based approach could be integrated with HCI methodologies that focus on usability and user engagement.

Klasnja et al.^7^ highlight the value of an HCI approach to interactive DHI development, arguing that HCI research should not be expected to consider the distal (e.g. behaviour change) outcomes, but should focus on proximal (interaction) outcomes. Klasnja et al.^7^ also note that a key value of HCI studies is that they can reveal why an interactive DHI did or did not work as intended. Smith et al.^8^ take a contrasting position, arguing that Klasnja et al.’s focus on proximal outcomes is unnecessarily limited. Smith et al. report on three studies that involved HCI researchers in the development of interactive DHIs. They argue that HCI offers substantive benefits in the development process for interactive DHIs, focusing on a ‘value chain’ of proximal, intermediate, and distal outcomes, where proximal outcomes are the immediately measurable effects such as user actions and perceptions, and distal outcomes are the longer-term effects on health.

### Evaluation

Murray et al.^1^ focus on the evaluation of interactive DHIs in terms of questions around health benefits and, conversely, risks of harm. More generally, much health-oriented research has been embedded in the paradigm of drug development and evaluation. Given the large sums of money involved in pharmacological research, and the well-acknowledged placebo effect, carefully designed RCTs that minimise the possibility of bias have become accepted as the ‘gold standard’ for determining the effectiveness of pharmacological agents. This approach has been transferred to evaluating non-pharmacological interventions, leading to a reification of the RCT as the primary method for evaluating all health care, and an acceptance of the ‘evidence pyramid’ with its hierarchy of evidence. This pyramid places well-conducted systematic reviews and meta-analyses of RCTs at the apex of the pyramid, followed by single RCTs, and then cohorts, case-control studies and, finally, case studies or opinions at the base^9,10^.

Many Health researchers have criticised this approach, and sought to develop new research methods for evaluating complex interventions. For example, Collins et al.^11^ adapt the classic RCT approach to evaluation of complex interventions to propose a multiphase optimisation strategy, which aims to simultaneously evaluate an intervention while iteratively optimising it through the process of development, fine-tuning and deployment of the intervention. Similarly, Mohr et al.^12^ summarise the limitations of traditional RCT methodologies for evaluating interactive DHIs, including the fact that the RCT does not permit iterative improvements to the design and that the technology may be outdated by the time the trial is complete. They propose that RCTs should focus on the evaluation of ‘intervention principles’ (i.e. the theoretical concepts represented within an interactive DHI) and suggest a methodology to achieve this. These, and other, novel methodologies tailored to the needs of interactive DHI development are essential, and welcomed. However, a full review of novel methods is beyond the scope of this paper. The dominant paradigm of ‘evidence-based medicine’ remains committed to RCTs, and it is essential for researchers working in this interdisciplinary space to understand this culture, if only because they are likely to encounter colleagues, reviewers or funding managers who espouse this perspective. For example, standards developed for reporting of interactive DHIs^13^ tend to focus on RCTs and not on alternative evaluation designs or formative research.

In contrast, Cresswell et al.^14^ note that there are many different kinds of questions that merit attention when evaluating interactive DHIs, and that the classic RCT is only applicable to a subset of those questions. They argue that safety and usability are important, and often necessary but not sufficient for effectiveness. They also note that the introduction of a new technology generally changes the work processes or social environment into which it is introduced, and that it is important to understand these effects too. They set HCI approaches in opposition to RCTs, in terms of the kinds of evaluation questions that each is best suited for.

The challenges of working across HCI (here, defined broadly to include software engineering) and Health are discussed by Pagliari^15^, who presents an account based on her own experience of interdisciplinary working across software engineering and Health. She identifies challenges as arising from non-shared concepts and language and incompatible values derived from scientific and technological research cultures with different historical roots, and describes researchers developing and evaluating interactive DHIs as working in ‘parallel universes.’ She summarises various lifecycle models that have been developed in both disciplines, highlighting the importance of iterative models, and proposes an overall lifecycle model based on three phases: evaluation of concepts and prototypes; evaluation of impacts; and pragmatic (post-deployment) evaluation. She argues that improved mutual understanding is essential for progress; we share this aspiration, identifying areas of contrast and commonality and reflecting on what we can learn from them.

### Implementation

The meaning ascribed to the word ‘implementation’ is an excellent example of how the two cultures use language differently and how confusion, or miscommunication, can easily arise. For HCI researchers, ‘implementation’ refers to the development of the computer programme (the digital intervention) that precedes many forms of evaluation. Here, implementation refers to operationalising and making tangible the design concepts that have emerged from understanding user requirements. In contrast, Health researchers would subsume this meaning of implementation into ‘development’. For them, ‘implementation’ refers to integrating an interactive DHI into routine health care, or otherwise ensuring wide-scale use across the target population. For Health researchers, implementation and ‘implementation science’^16^ are essential for closing the ‘second translational gap,’ or the gap between evidence (what should happen) and practice (what actually happens). As such, implementation is an activity that tends to occur after an intervention has been shown to be effective. In this paper, we use the word with both meanings, to avoid privileging one community over the other, and hope that the intended meaning is clear from the context. Because it is unlikely that two communities can converge on an agreed common meaning, we advocate interdisciplinary understanding rather than convergence on a common definition.

## Seven lessons

We present the complementary perspectives of HCI and Health in terms of seven lessons that interactive DHI researchers can learn from each area of expertise, based on key contrasts. These are summarised in Table 1, moving from how prior literature is reviewed (lesson 1), through the lifecycle of developing and testing a digital intervention and how “success” is measured, to how results are reported (lesson 7). These differences have developed for good reasons, based on history (as evolved through communities of practice) and values; each has its strengths and its limitations, such that each community can learn valuable lessons from the other. We present these as contrasts, or poles, while recognising that most practices related to interactive DHIs lie between the poles.

**Table 1.** Seven areas of contrast in practice between HCI and Health: the poles.

Lesson	Topic	HCI ‘pole’	Health ‘pole’
1	Establishing the state of the art (or what is known already from the literature)	Opportunistic (rarely structured).	Systematic.
2	Lifecycles	Iterative, focusing on fitness for purpose. Stages include: ascertaining user requirements, design, implementation (operationalising design concepts), and evaluation.	Iterative, focusing on impact. Stages include: development, feasibility and piloting, evaluation, and implementation (wide-scale deployment).
3	Requirements and design methods	End users are the primary ‘experts.’ A suite of methods for gathering user requirements and generating design solutions is employed to deliver a tool, application, or system. Emphasis is on creativity and innovation.	Clinicians and other professionals are the primary ‘experts.’ The focus is on the design of an ‘intervention,’ and the design process is encouraged to draw on theory. Emphasis on systematic development is based on ‘mechanisms of action.’
4	Implementation	Precedes evaluation; focuses on developing a computer system.	Follows evaluation; focuses on roll-out across care (or other) systems.
5	Evaluation methodologies and measures	Adapted from many disciplines; focuses on process.	RCT dominates; focuses on effects.
6	Ethics	Practice has historically focused more on individuals’ rights (through consent) than on risk of harm.	Highly regulated; the prevention of adverse events or harm is seen as a priority.
7	Publications	Difficult to publish anything other than basic research. Credible papers are rarely under 6000 words, and often over 10,000 words.	Variety of paper types including case notes, opinion pieces, letters, and research papers. Maximum word count is usually under 4000.

HCI, Human–Computer Interaction; RCT, randomised controlled trial.

In the following sections, we review each of these contrasts in practice, focusing particularly on what each community can usefully learn from the other.

## Lesson 1: Establishing the state of the art – what is known already

This is an area where HCI can be spectacularly scruffy and Health unhelpfully straight-laced. An HCI literature review tends to be an interesting meander through relevant and insightful literature that builds a case for a particular perspective. Historically, there are few known cases of wilful intellectual fraud, so HCI researchers have never felt a strong pressure to be systematic, rigorous and transparent in their approach to developing a literature review. In the days before good internet search engines and curated online resources, it was simply not possible to do a systematic review of HCI resources. Researchers could only search through particular journals, discover possible search terms through a process of trial and error, and undertake ad hoc backwards chaining (following trails of citations from one relevant paper back through the papers it cited to other relevant-looking papers). In contrast, Health research has historically focused on transparency, accountability, and the elimination of bias from reviews, and Health resources have been curated in a way that makes them comparatively easy to search consistently (the precursor to the MEDLINE indexing system, which has been online since 1971, was established as *Index Medicus* in 1869). Thus, the purest form of systematic review^17^ follows a ‘waterfall’ model: starting with clearly defined and fixed search terms and databases (maximising recall), applying a set of inclusion and exclusion criteria to improve search precision before systematic analysis of the remaining publications based on a pre-determined approach. Again, this methodology reflects the dominance of pharmacological research, and it works well for research questions focusing on the effectiveness of similar interventions in similar populations and contexts, with similar study methods. The limitations of this approach when the research question is not about effectiveness, or where the literature is methodologically heterogeneous, have been acknowledged, and novel review methodologies such as critical interpretive synthesis^18,19^, realist review^20^, and hermeneutic review^21,22^ have been developed and reported in recent years.

### Lessons to share

#### Lessons for interactive DHIs from HCI

HCI focuses on insight. It recognises that the researcher cannot know everything ahead of time. It values iteration and discovery, the construction of understanding, and sensemaking. It focuses on gathering evidence pertinent to the question at hand. Because it does not demand that the search be fully defined ahead of time, it is less daunting to start (particularly for early career researchers) than a systematic review. It covers a range of sources, including Computer Science journals that are typically missed from a Health-oriented search (because they are not indexed in Medline or similar).

#### Lessons for interactive DHIs from Health

Health focuses on systematicity, transparency and rigour. The approach is inspectable and reproducible (as long as it is clear on what date the original search was done). It aims to minimise researcher bias in the selection of papers for inclusion in a review, and account for bias in included papers. Students learning to conduct a systematic review will have gained a valuable research skill.

A good literature review achieves an appropriate balance between insight and rigour. Nunes et al.^2^ illustrate one approach to achieving this balance, drawing on the best of both cultures. Insight might focus on the individual researcher conducting the review (and their ‘journey’ towards better understanding of a domain). However, any review should also be of value to the research community, and should minimise bias; the value of comprehensiveness depends on the purpose of the review. Ultimately, reviews can serve multiple purposes, including:

- summative reviews that present the evidence for and against particular hypotheses or arguments;
- formative reviews that establish the state of the art (in terms of knowledge or methodology), on which a new study will build, or that identify gaps in the literature that can be filled by a future study.

Systematic reviews are better suited for the former, whereas more wide-ranging reviews that focus on insight are typically better suited to the latter. The ‘sweet spot’ generally lies between the extremes that are caricatured here. Researchers need to reflect on the purpose of the review that they are planning to conduct, and on what is possible given the existing literature, and choose the methodology accordingly.

## Lesson 2: Development lifecycles

**Figure 1.**
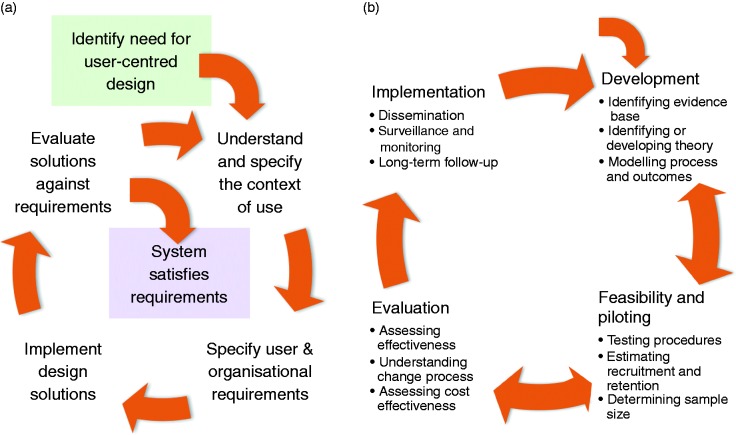
(a) The ISO 9241 HCI development lifecycle. (b) The MRC complex interventions development framework^4^. HCI, Human–Computer Interaction.

In terms of interactive DHIs, both HCI and Health have the aim, loosely stated, of developing and testing digital interventions to improve health. Both have evolved research and development lifecycles that encapsulate what is recognised as best practice within their area. Widely cited lifecycles from HCI and Health are summarised in Figure 1.

The HCI lifecycle^15^ starts with recognising a need for a new design (i.e. digital intervention), then focuses on understanding the context of use, specifying requirements, designing and implementing solutions, and then evaluating those solutions. This is an iterative process, which recognises that it is impossible to fully understand user needs up-front, and that user needs and practices evolve with new tools. Carroll and Rosson^23^ describe this in terms of a ‘task-artefact cycle’: As the design of artefacts changes, so do the activities around those artefacts, in ways that are both planned and unanticipated.

The Health lifecycle^4^ starts with development, ideally based on well-articulated theory (which encapsulates what is already known about the specific behaviour and needs of the target population) and draws on ‘mechanisms of action’ (i.e. what design features result in what behaviours through what underlying mechanisms), followed by pilot testing, evaluation (typically through an RCT), and implementation (i.e. roll-out to a broader population). As with the HCI lifecycle, the process is iterative – in principle, if rarely in practice. However, there is little consideration of the co-evolution of artefacts and activities.

Superficially, these cycles are similar: both are iterative and both involve evaluation and implementation. However, the similarities end there. For interactive DHIs, the entire HCI development lifecycle is subsumed within the first (‘Development’) phase of the MRC development framework.^4^ The other three phases of the MRC lifecycle focus on the testing of the intervention (through an RCT) and the roll-out of the intervention across different healthcare organisations (once it has been shown to be clinically effective).

### Lessons to share

#### Lessons for interactive DHIs from HCI

For interactive digital interventions, it is impossible to get the design right the first time. HCI has recognised this since at least 1986^24^. Thus, HCI has developed a variety of user-centred approaches for gathering user requirements, understanding the contexts in which systems will be used, and iteratively developing, testing and refining the design until it is fit for wide-scale deployment^25^. This deployment is referred to in Health as ‘implementation.’ Although investing resources up-front to develop a good design pays dividends, no design is ever complete; technology and technology-based practices evolve rapidly, so designs can always be improved.

#### Lessons for interactive DHIs from Health

Health focuses on the bigger picture, and particularly on health outcomes. Concerns extend beyond asking whether users accept the system and perceive it as being valuable, to asking whether the system is clinically- and cost-effective. Researchers in Health have developed frameworks and approaches such as MRC’s guidance on the development of complex interventions^4^ and the person-based approach to intervention development^6^, which make explicit the value of theory and the role of evidence in development that can get overlooked in HCI.

In ‘skating over’ the iterative development of interactive DHIs prior to any summative testing, and failing to recognise that development can be research at all, Health research takes two risks. The first is investing in large-scale evaluation studies of sub-optimally designed solutions; and the second is failing to learn about how the nuances of design affect user interaction and engagement (and hence health outcomes), such that success cannot be replicated in different contexts and failures risk being propagated from one design to another. Conversely, in focusing only on process and user perceptions (or process measures such as error rate or time to complete tasks), HCI fails to address the ultimate important question – namely, whether an interactive DHI is clinically effective. In practice, all of these questions are important.

## Lesson 3: Requirements and design methods

HCI has a repertoire of techniques for gathering and describing user requirements and designing interactive systems, including design patterns, scenarios, personas, task analysis and conceptual structuring^25^. Indeed, the development of novel and effective approaches for identifying requirements and developing designs is an active research topic in HCI. In addition, existing techniques being used by HCI consultants, and the development of interactive DHIs, present many opportunities for HCI research as well as practice.

HCI also has a tradition of ethnographic and other qualitative research^26^ that seeks to understand people’s needs and practices when they are using (or potentially using) interactive technologies. Although such studies often report ‘implications for design,’ their value in simply documenting practices and experiences, and developing theories of interactions and behaviours, is also recognised^27^. The practices around interactive DHI use and potential contexts of use offer a fertile area for HCI research.

Health typically focuses on theoretically derived ‘mechanisms of action’ or ‘active ingredients’ – terms and concepts that are derived from pharmacology research. There are comparatively few descriptions of how to gather requirements from users – largely because it is assumed that requirements are identified by professionals^28^. Thus, even when the identification of requirements is ‘person-based,’^6^ it is relatively unusual for the findings from qualitative user studies to be reported in their own right within Health, or to be regarded as being of comparable value to quantitative studies^29^. Conversely, there is a focus on developing and extending theory – e.g. theories of behaviour change or of decision making – to inform design.

This reflects an important but subtle difference in basic assumptions about the locus of expertise for which interactive DHIs are designed. HCI researchers have been trained to put the user at the centre ever since the early days of User Centred Systems Design^24^; furthermore, they work on the implicit assumption that the user is the expert in what they do and what they need^30^. Health researchers typically start with their own expertise, identifying a desired behaviour or clinical outcome; then, the challenge is to get the user of an intervention to engage, adhere, or comply with that intervention. The professional has a view on what is right, based on their expertise: a key challenge in designing an intervention is to make it palatable to the user so that they will engage with it^6^. Further, Health researchers place a value on their expertise: for example, they commonly employ techniques such as the Delphi method^31^, which is rarely found in HCI. This can create a conflict in views on who the ‘experts’ are, whose views should take precedence, and where requirements come from.

### Lessons to share

#### Lessons for interactive DHIs from HCI

The end-user has expertise that needs to be taken into account in design. The user is an expert in ‘being me’ and ‘managing my health conditions’ (amongst other things). Any interactive DHI that does not take the user’s social, physical, emotional, cognitive, individual situation, and expertise into account in design is destined to fail. This does *not* simply mean asking people what they want; although that gives an important perspective, ethnographic (and similar) research is needed to uncover people’s informal practices, tacit knowledge and motivating values.

#### Lessons for interactive DHIs from Health

The expertise of clinicians, psychologists and, indeed, HCI specialists has an important place – e.g. in motivation, sensemaking, myth-busting. This is often based on an accumulation of evidence of ways that people behave based on large-scale studies; for example, people aiming to quit smoking often believe that it is better to cut down gradually, whereas evidence from large-scale studies shows that people who quit in one step have a higher chance of success^32^.

Of course, neither community has the monopoly on the truth. Both have an important contribution to make. Any successful interactive DHI draws on all three kinds of expertise; that of clinicians, end-users, and HCI specialists. This includes expertise as encapsulated in existing theory and evidence from both HCI and Health.

There is a growing practice of co-design^33^ – i.e. involving end-users in design from an early stage. This gives end-users a stake in the design, and ensures that some users have a ‘voice’ in the design. However, it may raise questions about the representativeness of those co-designers: e.g. these individuals may be much more motivated than their peers. There is much still to do in developing approaches that represent end-user expertise without making heavy demands on potential end-users or involving self-selecting users who may not be representative of the broader population – particularly where the potential end-users are people with a substantial burden of self-management and who lack the skills to be effective co-designers. In designing interactive DHIs, there are situations in which the view that ‘we are all designers now’ may hold and others where the expertise of professionals is essential.

## Lesson 4: Implementation

As mentioned earlier, an area where HCI and Health language diverge is in what it means to ‘implement’ an interactive DHI. With its background in Computing, the HCI view of implementation is that it involves software development, and it places this form of implementation centre-stage in interactive DHI development. This step involves coding and testing (and typically follows or is interleaved with design). Where the implementation is well understood, employing established software development approaches, this is typically a ‘consultancy’ activity that can be outsourced to suitably qualified software developers. Where there are significant research challenges in the software development, such as the development and testing of novel machine learning algorithms for personalisation of an intervention (e.g. in Just In Time Adaptive Interventions)^34^ or of novel data privacy mechanisms, implementation becomes a research activity in its own right.

As described above, the Health view of implementation is that this is the final step, when a complex intervention (incorporating digital technologies) has been fully tested and is now being rolled out at scale. This view highlights an important point that has often been glossed over in HCI: namely that many interactive DHIs are part of a broader complex intervention, and that the broader care system may require re-design or re-configuration in order for the interactive DHI to be usable, safe, and effective.

### Lessons to share

#### Lessons for interactive DHIs from HCI

It is essential to focus necessary resources on software implementation to deliver an interactive DHI that works as designed – e.g. that data is not leaked to unauthorised people^35^, that algorithms work as intended, and that the software works reliably and safely.

#### Lessons for interactive DHIs from Health

If interactive DHIs are to be sustainable, and a good investment (of the time and resources of both individuals and care delivery systems), then it is essential to invest time and effort in ensuring that they are incorporated within broader care delivery systems in ways that work well for professionals, patients, and others involved in care.

Every interactive DHI has to be implemented in the sense that code needs to be written and tested; the complexity of the implementation and testing varies substantially across development projects, as do the requirements for research input into that process (depending on how established the necessary computing techniques are). The needs and practices for software testing also vary, depending on how complex and safety-critical the project is.

Some interactive DHIs (e.g. consumer products that are downloaded by individuals from an app store for their personal use) may not demand ‘implementation’ within a care system; nevertheless, they may have implications for that care system – e.g. as individuals’ approaches to managing their own health evolve in response to the new tools they have available to them. It may be impossible to plan for these changes ahead of time; more research on how interactive DHIs are adopted, adapted and used, and how they shape individual behaviour, social interactions and interactions between individuals and the care system (and care professionals), should improve our ability to anticipate and plan for the effects even when we cannot design them. This is an important area for future research.

## Lesson 5: Evaluation methodologies

Another area where HCI and Health practices diverge is in evaluation methodologies. Health evaluation typically focuses on effects and impact of an intervention. As noted above, the paradigmatic methodology is an RCT, which often involves extensive planning and large sample sizes (to achieve the statistical power necessary to detect clinically significant effects, which are typically small) and is hence expensive. RCTs are also time-consuming and, in the case of interactive DHIs, by the time the results are available and published, they may be obsolete. To limit the impact of confounding variables, the circumstances surrounding an RCT are controlled in ways that are not reflective of the range of natural user contexts. Whereas this may be necessary for drug trials, the simplifying assumptions that are necessarily made in an RCT are often inappropriate for interactive DHIs, limiting the real-world validity of such studies. Moreover, if there is no statistically significant effect, it is difficult to know whether the interactive DHI was sub-optimally designed and not fit-for-purpose or whether there were other reasons for the lack of measurable effect.

Conversely, in HCI most ‘evaluation’ is formative, testing features such as usability, usefulness and user experience. HCI evaluation can involve many different approaches to evaluation, including expert reviews of early prototypes^36^, lab studies involving think-aloud protocols of mature prototypes, and in-the-wild studies^26^ of robust implementations (i.e. studies that take place within the context of work, home or other natural settings). The importance of formative in-the-wild studies has been recognised for at least 30 years^37^.

### Lessons to share

#### Lessons for interactive DHIs from HCI

There are huge benefits in formative testing, drawing on multiple disciplines and practices. It is also important to test interactive DHIs in the final context of use (i.e. ‘in-the-wild’) – ideally, over an extended period of time. HCI research has identified a broad range of evaluation questions that are often summarised as ‘efficiency,’ ‘effectiveness,’ and ‘satisfaction.’ These process measures matter. Health funding rarely accommodates several stages of participatory or iterative design, but this is necessary for interactive DHIs.

#### Lessons for interactive DHIs from Health

While the RCT may have disadvantages, evaluation of clinical and cost-effectiveness is important. It must also be recognised that it is costly and time-consuming to evaluate effectiveness: HCI research timescales and funding mechanisms rarely accommodate this. Nevertheless, outcome measures matter.

**Figure 2.**
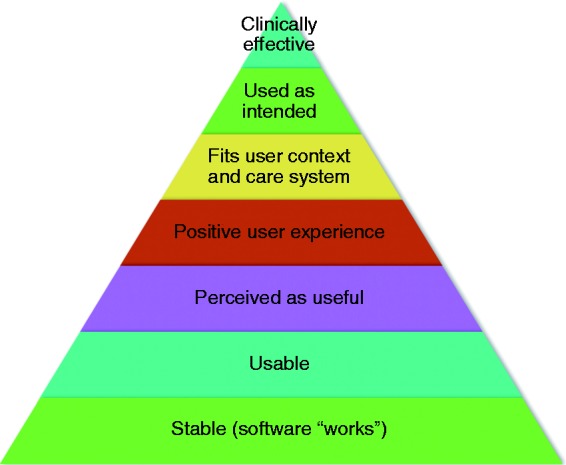
Dependencies between key classes of evaluation criteria for interactive DHIs: HCI (including software engineering) focuses on the lower levels, whereas Health focuses on the higher levels. HCI, Human–Computer Interaction.

Evaluation methods are (or should be) determined by the specific research questions posed. These questions have a logical partial ordering to them: usability relies on basic stability (i.e. no critical bugs); a system is unlikely to be useful if it is not usable; good user experience without usefulness is of little value for interactive DHIs (though it might be a fine quality of a computer game); system safety relies on stability and usability; etc. Clinical effectiveness is important, but is unlikely to be achieved without also establishing stability, usability, usefulness and fitness for purpose by people in their individual situations. Clinical and cost-effectiveness are of concern to clinicians and policymakers (first and foremost), although all the measures are of concern to end-users. This is discussed by Smith et al.^8^ in terms of proximal, intermediate, and distal outcomes. In Figure 2, we present this in terms of dependencies; each layer depends on the ones below.

## Lesson 6: Ethics/risk

HCI emerged from low-risk disciplines whereas any health intervention inevitably carries some risk, often exacerbated by inappropriate marketing of health-promoting products by ‘snake oil salesmen’^38^. Due to these different historical roots, HCI and Health have different attitudes to risk. In HCI, ethics focuses on the rights of the individual – as represented through informed consent to participation in studies. For example, there can be a lot of agonising about whether it is ethical to use social media records as data (because it is very difficult to obtain informed consent to use ‘posts’ after the fact)^39^. Although the importance of informed consent is recognised in Health, there is also an emphasis on potential harm to the participant. In the UK, the National Health Service (NHS) is particularly protective of patients, with a tendency to treat all studies as having the same potential for harm as an early stage drug trial. Thus, HCI studies involving interactive DHIs often seek ways to recruit participants without engaging with the NHS to avoid ethical processes that are seen as being disproportionate to the underlying risks of the study.

### Lessons to share

#### Lessons for interactive DHIs from HCI

In the context of interactive DHIs, the important expertise that a participant brings to a study is that of managing a clinical condition and using a particular technology. This does not necessarily make them particularly vulnerable, and hence at risk of harm from participating; indeed, assuming that someone is vulnerable simply because they are managing a health condition might be perceived as patronising, and it would be unethical to develop an interactive DHI without involving the intended users. Research that involves the design and evaluation of interactive DHIs needs to be considered on its own merits, in terms of the ethics of engaging participants.

#### Lessons for interactive DHIs from Health

Health highlights the importance of being respectful of people and of their time, particularly when they are managing a demanding clinical condition; it recognises that value comes from making a difference to people’s ability to self-manage and to their outcomes, and avoids the temptation to assume that people will engage in studies because they are interested. It also explicitly considers potential for harm, even for studies that are superficially low-risk.

Both areas place an emphasis on respect for participants. HCI tends to emphasise participants’ strengths (e.g. their expertise) whereas Health tends to emphasise their vulnerabilities and the risks of harm. Both are equally important considerations. A full discussion of ethics is outside the scope of this paper, but all researchers need to work within their disciplinary and local regulations as well as recognised best practices. This includes ensuring that they have considered all ethical issues pertinent to their study (e.g. when working with hard-to-reach groups, or managing sensitive data).

## Lesson 7: Publications and presentations

In HCI, there are two kinds of papers: conference papers and journal papers. Both are usually primary (archival) research. There may be higher- or lower-grade conferences and journals, but they all publish research papers, where that research is expected to be new, and to deliver insight. There may be the occasional review paper, but that is the exception rather than the rule.

In Health, there are many kinds of papers, but they are almost all published in journals; conferences typically publish abstracts, at most. As an example, the British Medical Journal lists 18 different kinds of papers, including ‘original research articles, review and educational articles, news, letters, investigative journalism, and articles commenting on the clinical, scientific, social, political, and economic factors affecting health’ (http://www.bmj.com/about-bmj/resources-authors, accessed 25 January 2018).

Two types of research papers that are common in Health but very rare (if ever seen) in HCI are the systematic review and the protocol paper. The first of these is discussed under Lesson 1, the particular point here being that these are commonly stand-alone papers with their own research question, methods and results, which should be reproducible. In contrast, in HCI, literature reviews are rarely stand-alone, and would normally constitute the background section of a full research paper. The second is unheard of in HCI but common in Health. There are two likely reasons for this; the first is that a protocol paper makes a clear, advance statement of how a study is going to be conducted and how data will be analysed, which creates a commitment, and accountability for the research team, and reduces the likelihood of ‘mission drift’ or ‘p-hacking’^40^ in a study; the second is differences in word limits. HCI research papers are almost invariably self-contained, including all relevant details of background, methods, results and discussion within the one paper; Health papers may focus principally on one of these components.

Word count: under or over 4000 words? Health papers are, almost without exception, under 4000 (or even lower limits). For HCI, this would be considered very short, and papers of over 10,000 words are common. Where Health papers are self-contained, this necessarily means that they are concise. But they may well contain substantial tables of essential details. For example, illustrative quotations generally appear in a table in a Health paper whereas they are integrated into the narrative in an HCI paper. Thus, the differences in word limits are not, in practice, necessarily as great as the headline figures suggest.

There is also a cultural difference in review processes: in HCI, journal papers are rarely rejected without review, and are more commonly ‘revise and resubmit’ than rejected even after first review. Of course, conference papers experience (pretty much) straight acceptance or rejection because of the different timescales that are possible. In contrast, Health journals often desk-reject papers early on, or reject completely after first review. Strategically, this means that an approach that is common in HCI of submitting papers when they are ‘good enough’ with the expectation that reviewers will help to make them better can be ‘instant reject’ in Health journals.

There are also differences in the order of authors, as discussed by Tscharntke et al.^41^: in HCI, authors are most commonly listed in order of contribution (so the second author position is more important than the last author), whereas in Health the team leader is typically listed last, reducing the status of all authors between first and last. Although it might be tempting to exploit this difference (list the HCI lead second and the Health lead last), it risks perpetuating perceptions amongst readers that the ‘own area’ lead contributed more to the publication than the ‘other area’ lead.

In HCI, the culture around conference presentation is that all (except a very small number of keynote speakers) will have submitted a full paper, and that paper is, by default, archival. Conferences that select presenters based on abstracts are second-tier, of questionable quality. Consequently, full HCI conference papers are highly regarded and often well cited, and the best conferences are considered of comparable quality to good journal publications. In contrast, in Health, conference presentations are most commonly by invitation, or based on the submission of an abstract. This can make it difficult for people from one area to present at conferences organised for and by the other.

### Lessons to share

#### Lessons for interactive DHIs from HCI

If you are aiming for insight and coherence, there is a lot to be said for including all the necessary information, in a natural order, within one paper. Academic conferences serve an important role in knowledge exchange and advancing the state of the art. Particularly in fast-moving areas based around technology research (including interactive DHIs), there is value in archival conference proceedings based on the submission of full papers where what is presented is worthy of publication (and citation).

#### Lessons for interactive DHIs from Health

There is huge value in various kinds of publications. For example, it is unusual to have ‘methods’ papers published in HCI journals, and yet these are essential for advancing the community and establishing best practices in research. The many other kinds of publications that are found in Health journals serve valuable roles – as long as it is clear what is basic research and what is commentary of some kind. There is a lot to be said for succinctness, and for publications that focus on key messages.

The take-home message for any researcher is that they have to understand the culture of the journal or conference they are aspiring to publish in, and work with the practices of that community. This is most easily achieved by working in interdisciplinary teams where each team member can learn from others.

## Discussion

### A more general lesson

The differences in cultures, practices and assumptions that we have highlighted here are subtle yet pervasive, and can easily trip up researchers who are unaware of the differences. Although we have focused on the positive – on what each area can learn from the other – there is also a challenge: that addressing an audience from ‘the other side’ as if you are on the same side can result in incomprehension and rejection. The Health researcher is likely to regard HCI research as scruffy, unsystematic, and unreliable. The fact that it does not focus on clinical outcomes limits its value. Conversely, the HCI researcher is likely to regard Health research as pedestrian and lacking insight or creativity. The lack of attention to process and producing the best possible design of an interactive DHI that really addresses the end-user’s individual situation means that it risks delivering inadequate, paternalistic digital interventions. Further, the focus on population level needs means that Health interventions are often designed for the ‘average’ user, whereas HCI recognises individual differences and market segments.

Until there is much greater mutual understanding and mutual valuing of the complementary research traditions than exists at present, people risk disappointment and rejection in trying to bridge the divide. Apply to a health funder for developmental work without any plan for a clinical trial? Forget it! Apply to an engineering funder for a summative evaluation of a novel health technology? Out of scope! Siloed research funding does not accommodate the exploration and iteration that are essential to develop effective interactive DHIs. Funders, policymakers and other organisations with substantial influence in this space need to understand the challenges that researchers face, and facilitate the joined-up thinking that was articulated by Pagliari^15^ over a decade ago.

Try to publish HCI research in a Health journal to improve the impact of the research or the results of a summative evaluation in an HCI journal? Low chances of success, and even lower chances of the paper being cited even if it is published. And if you are a PhD student working across this divide, you have a huge amount to learn from the other discipline – but take care in selecting examiners.

## Conclusion

This paper conforms with the HCI stereotype in aiming for insight over rigour and with the Health stereotype of drawing on our own expertise. We have focused on the ‘poles’ to communicate the contrasts that may be experienced. We also recognise that cultures and practices are converging – that the lessons that we highlight here are being learned by groups across the globe who are experiencing the same need to work across disciplines to develop interactive DHIs that are effective at all levels (Figure 2), and that new approaches and methodologies are being developed to address the lessons identified here. A review of these novel approaches is beyond the scope of this paper. Our aim in articulating these lessons is to help other researchers preparing to navigate this interdisciplinary space and, most importantly, to facilitate working together to improve the quality and utility of future research in interactive DHIs.
